# 中国成人早期前体T细胞急性淋巴细胞白血病诊断与治疗专家共识（2023年版）

**DOI:** 10.3760/cma.j.issn.0253-2727.2023.12.002

**Published:** 2023-12

**Authors:** 中国抗癌协会血液病转化医学专业委员会 中华医学会血液学分会淋巴瘤学组 中国女医师协会血液学专业委员会

T细胞急性淋巴细胞白血病（T-cell acute lymphoblastic leukemia，T-ALL）是一类具有高度侵袭性的淋巴细胞恶性增殖性疾病，占成人急性淋巴细胞白血病的20％～25％[1]。早期前体T细胞（Early T cell precursor，ETP）急性淋巴细胞白血病（ETP-ALL）是近年来提出的T-ALL的一类特殊亚型[2]。与其他T-ALL相比，ETP-ALL具有独特的免疫表型以及特殊的分子生物学特征[2]–[5]。为了进一步提高对该疾病的认识，规范诊断和治疗，中国抗癌协会血液病转化医学专业委员会、中华医学会血液学分会淋巴瘤学组和中国女医师协会血液学专业委员会组织国内血液学相关专家制定了此共识。

一、概述

ETP细胞是造血祖细胞由骨髓进入胸腺后定植在胸腺中的最早的祖细胞[6]，具有分化为T细胞和NK细胞的潜能[7]。2008年，Nature杂志同期发表了2篇研究论文提出ETP具有髓系分化的潜能[8]–[9]。2009年，美国St. Jude儿童医院的研究人员在T-ALL患儿中发现12.6％的患者白血病细胞具有ETP细胞的特征，这类患者具有独特的免疫表型，基因表达谱不同于T-ALL，长期生存率显著低于其他T-ALL患者，由此，他们将这类特殊的T-ALL命名为ETP-ALL[2]。2016年ETP-ALL获得WHO正式命名。ETP-ALL是T-ALL的一类特殊亚型，占成人T-ALL的12.6％～47.6％[2],[10]–[13]，男性多于女性，中位发病年龄29～40岁[10],[13]–[15]。国内报道的ETP-ALL占成人T-ALL的比例为17.3％～47.6％[14],[16]，略高于国外的12.6％～32％[10],[13],[17]，可能与种族差异有关。与经典T-ALL相比，ETP-ALL患者发病年龄较大，发病时白细胞计数较低[10],[14]，中枢神经系统浸润发生率较传统T-ALL高（16％对3％）[13]。

二、ETP-ALL的基因表达特点

ETP-ALL起源于胸腺中的ETP细胞。ETP细胞不仅具有分化为T细胞和NK细胞的潜能[7]，同时具有髓系分化潜能[8]–[9]。ETP-ALL基因表达谱也与经典T-ALL存在显著差异，除了T-ALL中常见的突变如ETV6、NOTCH1、DNM2外[18]，髓系白血病相关基因突变如FLT3、DNMT3A、RUNX1等在ETP-ALL中发生率更高[3]–[4], [18]。表1详细汇总了ETP-ALL基因表达谱的特点。相较于其他类型的T-ALL，ETP-ALL主要存在以下分子遗传学特点：①细胞因子受体/RAS信号通路相关基因突变较典型T-ALL多见（如NRAS、KRAS、FLT3、IL7R、JAK3、JAK1、SH2B3、BRAF）[3],[10]；②造血细胞发育相关基因突变较典型T-ALL多见（如GATA3、ETV6、RUNX1、IKZF1、EP300等）[4],[10]；③组蛋白修饰相关基因突变较典型T-ALL多见（如EZH2、EED、SUZ12、SETD2、EP300等）[4],[10]；④DNA甲基化相关基因突变如DNMT3A、IDH1、IDH2、TET2、TET3、WT1等较典型T-ALL多见[10],[18]。

**表1:** 早期前体T细胞急性淋巴细胞白血病（ETP-ALL）中分子生物学改变特点[3]–[4],[10],[17]–[18]

基因	发生率（ETP-ALL对非ETP-ALL）	与非ETP-ALL差异
细胞因子受体/RAS通路相关基因（NRAS、KRAS、FLT3、IL7R、JAK3、JAK1、SH2B3、BRAF等）	62.2%~67.2%对19%~37.8%	显著高于非ETP-ALL
造血细胞发育相关基因（GATA3、ETV6、RUNX1、IKZF1、EP300等）	29.7%~57.8%对11.9%~16.7%	显著高于非ETP-ALL
组蛋白修饰相关基因（EZH2、EED、SUZ12、SETD2、EP300等）	42.2%~48.6%对11.9%~29.6%	显著高于非ETP-ALL
DNA甲基化相关基因（DNMT3A、IDH1、IDH2、TET2、TET3、WT1等）	32.4%对23.7%	略高于非ETP-ALL
其他重要的基因改变		
NOTCH1/FBXW7突变	14%~53.2%对70.5%	低于非ETP-ALL
MLLT10易位	21.3%对3%	显著高于非ETP-ALL
TLX1	0对27.1%	显著低于非ETP-ALL
HOXA	50%对19.6%	显著高于非ETP-ALL

三、ETP-ALL的诊断及鉴别诊断

（一）ETP-ALL的诊断

ETP-ALL的诊断标准参照WHO 2016淋巴和造血组织肿瘤分类标准[5]：在诊断为T-ALL的基础上，原始细胞免疫分型需满足以下条件：①CD7阳性，CD1a和CD8阴性。②CD5阴性或阳性率<75％。③至少一项髓系/干细胞抗原标志阳性，包括CD34、CD117、HLA-DR、CD13、CD33、CD11b或CD65；MPO阴性。④典型的ETP-ALL可表达CD2和胞质CD3，可表达CD4，但不作为诊断要求[5]。

日常诊疗工作中，为了更好地识别ETP-ALL，也可参照ETP-ALL诊断积分系统[19]（表2）。11项免疫标志包括：CD1a、sCD3（胞膜CD3）、CD5、CD8、CD10、CD13、CD33、CD34、CD117、TdT和MPO。其中MPO表达≥3％为MPO阳性，CD5表达≥75％为CD5阳性，其余标志均以表达≥20％判断为阳性。分数相加，积分≥8分诊断为ETP-ALL，积分<8分为其他类型T-ALL。

（二）ETP-ALL的鉴别诊断

1. T/髓混合细胞白血病：T/髓混合细胞白血病与ETP-ALL均可表达T-ALL标志及髓系标志，因此容易混淆。根据WHO 2016淋巴和造血组织肿瘤分类标准[5]，T/髓混合细胞白血病的诊断需同时满足髓系及T细胞系的要求，其中髓系要求MPO阳性或单核细胞分化（至少具备以下中两条：NSE、CD11c、CD14、CD64、溶菌酶），T细胞系要求CD3表达（胞质或表面）。ETP-ALL可满足T细胞系的要求，但不符合前述髓系要求，可作为二者的鉴别。

**表2:** 早期前体T细胞急性淋巴细胞白血病（ETP-ALL）诊断积分系统[19]

分子标志	表达	不表达
CD1a	−2	2
sCD3	−2	
CD5	−2	2
CD8		2
CD10		1
CD13	1	
CD33	1	
CD34	1	
CD117	1	
TdT		1
MPO	−3	

注 ETP-ALL通常积分≥8分，而其他类型T-ALL积分<8分

2. Near ETP-ALL：near ETP-ALL同样表现为CD7阳性，CD1a和CD8阴性，MPO阴性，同时表达髓系或干细胞抗原标志，但near ETP-ALL强表达CD5，而ETP-ALL弱表达CD5或CD5阴性，是二者主要区别[20]。Near ETP-ALL虽然与ETP-ALL免疫表型相似，但临床特点与非ETP-ALL更相似，主要表现为常规化疗方案下near ETP-ALL预后与非ETP-ALL相似，优于ETP-ALL，另外，首次完全缓解（CR_1_）后异基因造血干细胞移植可显著改善ETP-ALL患者预后，而near ETP-ALL及非ETP-ALL患者无显著获益[21]。

3. 典型T-ALL：ETP-ALL是T-ALL的一类特殊亚型，具有特殊的免疫表型，临床工作中与其他非ETP-ALL的鉴别尤为重要。ETP-ALL与典型T-ALL，包括Pro-T-ALL、Pre-T-ALL、皮质（胸腺）T-ALL、髓质（成熟）T-ALL的免疫表型比较见表3。根据表3所示，ETP-ALL与皮质T-ALL、髓质T-ALL容易鉴别。皮质T-ALL同时表达CD4和CD8，且CD1a阳性，而ETP-ALL CD8阴性、CD1a阴性，可与ETP-ALL鉴别。髓质T-ALL膜表面CD3（sCD3）阳性，可与ETP-ALL鉴别。而对于Pro-T-ALL和Pre-T-ALL，两种T-ALL亚型均符合CD1a阴性、CD8阴性、CD7阳性，因此新提出的ETP-ALL亚型包含于该两类传统T-ALL分类之中。最终是否诊断为ETP-ALL还需进一步根据CD5表达水平以及是否表达髓系/干细胞抗原标志而确定。

**表3:** 不同T细胞急性淋巴细胞白血病（T-ALL）亚型的免疫分型[5],[21]

亚型	CD1a	CD2	cCD3	sCD3	CD5	CD4	CD8	CD7	髓系/干系
Pro-T	−	−	+	−	−	−	−	+	+/−
Pre-T	−	+	+	−	+	−	−	+	+/−
皮质T（胸腺T）	+	+	+	+/−	/	+	+	+	−
髓质T（成熟T）	−	+	+	+	/	CD4+或者CD8+，不会同时阳性	+	−
ETP-ALL	−	+/−（典型ETP-ALL阳性）	+/−（典型ETP-ALL阳性）	−	<75%	+/−	−	+	+

注 ETP-ALL：早期前体T细胞急性淋巴细胞白血病

四、ETP-ALL的治疗

患者一经确诊应尽快开始治疗。ETP-ALL的治疗分为诱导治疗（部分病例需要预治疗）、缓解后的巩固强化治疗、维持治疗等几个阶段及髓外白血病（中枢系统白血病）的预防和治疗。

（一）诱导治疗

1. 治疗原则：①首选临床试验；②多药联合化疗方案。

2. 治疗方案：参加临床试验者依照试验方案进行，不适合或无法参加临床试验者诱导治疗以联合化疗为主[22]，化疗方案参照目前Ph染色体阴性的B-ALL。

多药联合化疗推荐方案：

（1）中国成人急性淋巴细胞白血病协作组（CALLG）——CALLG2008治疗方案[23]。

（2）Hyper-CVAD方案（MDACC）：Kantarjian H. Cancer, 2004, 101: 2788-2801[24]。

（3）GRAALL-2005方案：Huguet F. J Clin Oncol, 2018, 36:2514-2523[25]。

（4）CALGB8811方案：Larson RA. Blood,1995, 85（8）: 2025-2037[26]。

ETP-ALL对糖皮质激素治疗不敏感，预治疗不推荐糖皮质激素，建议白细胞去除术或小剂量阿糖胞苷预治疗。以上常规诱导治疗方案缓解率不高，缓解深度不够，1个疗程化疗后微小残留病（MRD）阳性率高[2],[10],[13],[27]。因此ETP-ALL诱导治疗方案仍在不断探索和完善中。对于初发ETP-ALL患者，强烈推荐参加临床试验，临床试验方案总结见表4。

**表4:** 早期前体T细胞急性淋巴细胞白血病（ETP-ALL）新型治疗药物/方案汇总[30]–[42]

新治疗	理论基础	治疗靶标	单药治疗/合并治疗	是否进入临床试验阶段
BCL-2抑制剂（维奈克拉）	ETP-ALL相对于非ETP-ALL表达更高水平的BCL-2[31]–[34]	BCL-2	联合化疗	是（初发和复发难治）
JAK抑制剂（芦可替尼）[35]	ETP-ALL中JAK/STAT通路激活	JAK	1. 单药 2. 联合化疗	是（复发难治）
去甲基化药物（地西他滨/阿扎胞苷）	DNA甲基化相关基因如DNMT3A在ETP-ALL高表达	表观遗传学相关基因改变	联合化疗	是（初发）
组蛋白去乙酰酶抑制剂（西达本胺）[36]	ETP-ALL中组蛋白修饰相关基因异常高表达	组蛋白修饰相关基因	联合化疗[37]	是（初发）
CAR-T细胞[38]	制备CAR-T细胞攻击ETP-ALL细胞[39]	CD5、CD7[40]	单药	否
FLT3抑制剂	在ETP-ALL中FLT3突变率高[10]	FLT3	单药[41]	否
CD33偶联抗体	部分ETP-ALL患者存在CD33高表达[19]	CD33	单药	否
CD38抗体（达雷妥尤单抗）[42]	ETP-ALL患者存在CD38高表达	CD38	1. 单药 2. 联合奈拉滨	否

3. 注意事项：

（1）预处理：对于WBC≥30×10^9^/L或肝、脾、淋巴结明显肿大者，建议进行预治疗，以防止肿瘤溶解综合征的发生。预处理方案：ETP-ALL患者对糖皮质激素治疗不敏感，建议采用小剂量阿糖胞苷或羟基脲进行预处理。或采用白细胞去除术。

（2）尽早开始腰椎穿刺进行鞘内注射（鞘注）治疗，预防中枢神经系统白血病（CNSL）的发生，建议在血小板计数达安全水平、外周血没有原始细胞时进行。

（二）CR后的治疗

1. 治疗原则：青少年患者和年龄<60岁的成人患者，建议CR后进行异基因造血干细胞移植，如果无移植条件，继续多药联合化疗；≥60岁体能状态好的患者可考虑减低剂量预处理或常规预处理的异基因造血干细胞移植；不适合强烈治疗者（高龄、体能状态较差、严重脏器合并症等）可考虑低强度联合化疗巩固。

2. 治疗方案：

（1）异基因造血干细胞移植可显著改善ETP-ALL患者的长期生存[10],[15]，建议在CR_1_期就进行异基因造血干细胞移植[28]–[29]。

（2）如果无移植条件或等待移植的患者，缓解后巩固治疗可清除残存的白血病细胞，提高疗效。缓解后治疗方案推荐如下：

a. 对于诱导治疗参加临床试验的患者，可按照临床试验序贯方案进行；

b. 对于诱导治疗采用CALLG2008治疗方案、Hyper-CVAD方案（MDACC）、GRAALL-2005方案、CALGB8811方案等序贯方案的患者，可采用原序贯方案中的巩固治疗方案；

c. 推荐含阿糖胞苷的巩固治疗方案。

3. 注意事项：

（1）为减少复发、提高生存率，诱导治疗结束后应尽快开始缓解后的巩固强化治疗，间歇期不宜过长。

（2）ETP-ALL患者中枢神经系统浸润发生率较高，已达CR的患者均建议行腰椎穿刺、鞘注化疗。脑脊液中如发现白血病细胞，应每周2次鞘注化疗直至脑脊液正常，以后每周1次持续4～6周。对于没有CNSL的患者建议进行4次鞘注化疗预防。鞘注治疗药物同B-ALL。

（三）维持治疗

对于无条件进行异基因造血干细胞移植的患者强调维持治疗，目前ETP-ALL的维持治疗尚无统一方案，可参考Ph染色体阴性的B-ALL维持治疗方案。

（四）复发难治ETP-ALL的治疗

复发难治ETP-ALL的治疗目前无统一意见，首先推荐临床试验，临床试验方案及化疗方案推荐如下：

1. 维奈克拉+HAG方案（浙江大学医学院附属第一医院，注册号ChiCTR2100048966）：维奈克拉+高三尖杉酯碱+小剂量阿糖胞苷+G-CSF。

2. 芦可替尼+LVP方案（四川大学，注册号NCT03613428）：芦可替尼+门冬酰胺酶+长春地辛+地塞米松。

3. 可选择未用过的强化方案，如未接受过Hyper-CVAD方案的复发难治患者可以选择该方案，也可选择含克拉曲滨或氟达拉滨的强化方案。

对于无法耐受以上强化疗方案的患者，也可根据患者具体情况采用FLT3抑制剂，CD38单抗等单药或联合化疗的治疗方案，复发难治ETP-ALL的临床试验方案总结见表4。另外，无论复发还是难治的ETP-ALL患者，在挽救治疗开始即应考虑造血干细胞移植，及时寻找供者，一旦缓解尽快实施异基因造血干细胞移植。

五、预后

按照传统化疗方案，ETP-ALL的缓解率低，预后较差，无事件生存（EFS）时间及总生存（OS）时间均短于非ETP-ALL患者[2],[13]。其原因主要是ETP-ALL对常规化疗方案中糖皮质激素及化疗药物耐药发生率明显高于非ETP-ALL（63.8％对36.8％；87.0％对33.7％）[10]，从而导致缓解质量差，MRD阳性率也高于非ETP-ALL（71.4％对20.9％）[10],[13]，容易发生难治或者复发。但是，如能增加化疗强度，并根据MRD及时调整治疗，达到CR/CRi后尽早进行异基因造血干细胞移植则可扭转ETP-ALL患者的较差预后，长期生存也能达到非ETP-ALL患者的水平[10], [43]–[44]。

六、总结

ETP-ALL是近年来新提出的一类T-ALL亚型，具有特殊的免疫表型和独特的基因表达谱，主要表现为髓系相关基因突变发生率更高。ETP-ALL对常规ALL的一线诱导化疗方案疗效不佳，临床试验应作为首选的治疗方案，ETP-ALL患者获得CR后应尽早进行异基因造血干细胞移植，可以显著改善患者的生存预后。随着精准医学时代的到来，联合新的靶向药物治疗是未来能进一步改善ETP-ALL患者生存的新方向。
